# Supplementary material for: Higher Accuracy of Lung Ultrasound over Chest X-ray for Early Diagnosis of COVID-19 Pneumonia
Source: Int J Environ Res Public Health. 2021 Mar 27;18(7):3481. doi: 10.3390/ijerph18073481 (PMC8037158; doi:10.3390/ijerph18073481)
Supplement: Supplementary file 1 [file ijerph-18-03481-s001.zip › supplementary files/Back matter.pdf]

1. Supplementary Materials

Supplementary Table 1. Contingency table of fever and pathologic chest X-ray findings.

Fever	Pathologic X-ray	RT-PCR +	RT-PCR-
Yes	Yes	41	9
No	No	5	37
No	Yes	6	5
Yes	No	35	49

Supplementary Table 2. Contingency table of fever and pathologic findings with a diffuse pattern by lung ultrasound.

Fever	Diffuse pattern	RT-PCR +	RT-PCR-
Yes	Yes	50	4
No	No	10	43
No	Yes	4	2
Yes	No	37	62

Supplementary Table 3. Contingency table of fever and pathologic findings with a local pattern by lung ultrasound.

Fever	Local pattern	RT-PCR +	RT-PCR-
Yes	Yes	17	18
No	No	8	37
No	Yes	6	8
Yes	No	70	48

Supplementary Table 4. Contingency table of fever and normal lung ultrasound findings.

Fever	Normal ultrasound	PCR +	PCR-
Yes	Yes	20	44
No	No	10	10
No	Yes	4	35
Yes	No	67	22

2. Acknowledgments

The authors would like to thank the all patients who participate in the study, all Professionals in the Health Care Center Balaguer, to the Consell Comarcal de la Noguera, Balaguer, Spain . The authors also would like to thank Angela Rynne Vidal, PhD, and her colleagues from The Paper Mill for providing writing support.

3. Author Contributions

JMR, MOB, JMPP and JPS conceived the study design and oversaw the project. JMR, MCP, CGS were responsible for data acquisition. CCR performed the statistical analyses.

All authors read and approved the final version of this manuscript.

4. Institutional Review Board Statement

This study was approved by the Ethical Committee of Institut d'Investigació en Atenció Primària Jordi Gol i Gurina (Barcelona, Spain) (registration number p20/138).

5. Informed Consent Statement

Informed consent was obtained from all subjects involved in the study.

6. Conflicts of Interest

The authors declare no competing interests.

7. References

1. Hiscott J, Alexandridi M, Muscolini M, et al. The global impact of the coronavirus pandemic. *Cytokine Growth Factor Rev* 2020; 53: 1–9.

2. WHO. Archived: WHO Timeline - COVID-19, https://www.who.int/news/item/27-04-2020-who-timeline---covid-19 (accessed 1 January 2021).

3. Spain Ministry of Health. COVID-19 update number 41, March 11, 2020, https://www.mscbs.gob.es/profesionales/saludPublica/ccayes/alertasActual/nCov/documentos/Actualizacion_41_COVID-19.pdf (accessed 20 January 2021).

4. Spain Ministry of Health. COVID-19 update number 60, March 31, 2020, https://www.mscbs.gob.es/profesionales/saludPublica/ccayes/alertasActual/nCov/documentos/Actualizacion_61_COVID-19.pdf (accessed 20 January 2021).

5. Guan W, Ni Z, Hu Y, et al. Clinical Characteristics of Coronavirus Disease 2019 in China. *N Engl J Med* 2020; 382: 1708–1720.

6. Esakandari H, Nabi-Afjadi M, Fakkari-Afjadi J, et al. A comprehensive review of COVID-19 characteristics. *Biol Proced Online*; 22. Epub ahead of print 2020. DOI: 10.1186/s12575-020-00128-2.

7. Jiménez E, Fontán-Vela M, Valencia J, et al. Characteristics, complications and outcomes among 1549 patients hospitalised with COVID-19 in a secondary hospital in Madrid, Spain: A retrospective case series study. *BMJ Open*; 10. Epub ahead of print 2020. DOI: 10.1136/bmjopen-2020-042398.

8. Li R, Rivers C, Tan Q, et al. The demand for inpatient and ICU beds for COVID-19 in the US: lessons from Chinese cities. *medRxiv Prepr Serv Heal Sci*. Epub ahead of print 2020. DOI: 10.1101/2020.03.09.20033241.

9. Henríquez J, Gonzalo-Almorox E, García-Goñi M, et al. The first months of the COVID-19 pandemic in Spain. *Heal Policy Technol* 2020; 9: 560–574.

10. Calvo-Cebrián A, Alonso-Roca R, Rodriguez-Contreras FJ, et al. Usefulness of Lung Ultrasound Examinations Performed by Primary Care Physicians in Patients With Suspected COVID-19. *J Ultrasound Med*. Epub ahead of print 2020. DOI: 10.1002/jum.15444.

11. Axell-House DB, Lavingia R, Rafferty M, et al. The estimation of diagnostic accuracy of tests for COVID-19: A scoping review. *Journal of Infection*, 2020, pp. 681–697.

12. Vitiello A, Pelliccia C, Ferrara F. COVID-19 Patients with Pulmonary Fibrotic Tissue: Clinical Pharmacological Rational of Antifibrotic Therapy. *SN Compr Clin Med* 2020; 2: 1709–1712.

13. Spanish Society of Medical Radiology. *Basic guide of indications for imaging tools for COVID-19 infection*, https://www.seram.es/images/site/Recomendaciones_imagen_SERAM_COVID_19.pdf (2020).

14. Cozzi A, Schiaffino S, Arpaia F, et al. Chest x-ray in the COVID-19 pandemic: Radiologists' real-world reader performance. *Eur J Radiol*; 132. Epub ahead of print 2020. DOI: 10.1016/j.ejrad.2020.109272.

15. Schiaffino S, Tritella S, Cozzi A, et al. Diagnostic Performance of Chest X-Ray for COVID-19 Pneumonia during the SARS-CoV-2 Pandemic in Lombardy, Italy. *J Thorac Imaging* 2020; 35: W105–W106.

16. Kim HW, Capaccione KM, Li G, et al. The role of initial chest X-ray in triaging patients with suspected COVID-19 during the pandemic. *Emerg Radiol* 2020; 27: 617–621.

17. Gargani L, Picano E. The risk of cumulative radiation exposure in chest imaging and the advantage of bedside ultrasound. *Crit Ultrasound J*; 7. Epub ahead of print 2015. DOI: 10.1186/s13089-015-0020-x.

18. Sorensen B, Hunskaar S. Point-of-care ultrasound in primary care: a systematic review

of generalist performed point-of-care ultrasound in unselected populations. *Ultrasound J*; 11. Epub ahead of print 2019. DOI: 10.1186/s13089-019-0145-4.

19. Volpicelli G, Elbarbary M, Blaivas M, et al. International evidence-based recommendations for point-of-care lung ultrasound. In: *Intensive Care Medicine*. Springer, pp. 577–591.

20. Volpicelli G, Gargani L. Sonographic signs and patterns of COVID-19 pneumonia. *Ultrasound J*. Epub ahead of print 2020. DOI: 10.1186/s13089-020-00171-w.

21. Lindgaard K, Riisgaard L. Validation of ultrasound examinations performed by general practitioners. *Scand J Prim Health Care* 2017; 35: 256–261.

22. Solanes Cabus M, Pujol Salud J, Alonso Aliaga J, et al. Inter-rater agreement and reliability among General Practitioners and Radiologists on Ultrasound examinations after specialized Training Program. *J Prim Heal Care Gen Pract*.

23. Zieleskiewicz L, Markarian T, Lopez A, et al. Comparative study of lung ultrasound and chest computed tomography scan in the assessment of severity of confirmed COVID-19 pneumonia. *Intensive Care Med* 2020; 46: 1707–1713.

24. Lu W, Zhang S, Chen B, et al. A Clinical Study of Noninvasive Assessment of Lung Lesions in Patients with Coronavirus Disease-19 (COVID-19) by Bedside Ultrasound. *Ultraschall der Medizin* 2020; 41: 300–307.

25. Pare JR, Camelo I, Mayo KC, et al. Point-of-care lung ultrasound is more sensitive than chest radiograph for evaluation of COVID-19. *West J Emerg Med* 2020; 21: 771–778.

26. Haak SL, Renken IJE, Jager LC, et al. Diagnostic accuracy of point-of-care lung ultrasound in COVID-19. *Emerg Med J*. Epub ahead of print 2020. DOI: 10.1136/emermed-2020-210125.

27. Sorlini C, Femia M, Nattino G, et al. The role of lung ultrasound as a frontline diagnostic tool in the era of COVID-19 outbreak. *Intern Emerg Med*. Epub ahead of print 2020. DOI: 10.1007/s11739-020-02524-8.

28. Emanuele P, Alberto G, Maria T, et al. Lung ultrasound for the diagnosis of SARS-CoV-2 pneumonia in the Emergency Department. *Ann Emerg Med*. Epub ahead of print 2020. DOI: 10.1016/j.annemergmed.2020.10.008.

29. Casas-Rojo JM, Antón-Santos JM, Millán-Núñez-Cortés J, et al. Clinical characteristics of patients hospitalized with COVID-19 in Spain: Results from the SEMI-COVID-19 Registry. *Rev Clin Esp* 2020; 220: 480–494.

30. Hon KL, Leung KKY, Leung AKC, et al. Overview: The history and pediatric perspectives of severe acute respiratory syndromes: Novel or just like SARS. *Pediatric Pulmonology*, 2020, pp. 1584–1591.

31. Trauer M, Matthies A, Mani N, et al. Utility of Lung Ultrasound in COVID-19: A Systematic Scoping Review. *Ultrasound* 2020; 2020.06.15.20130344-

2020.06.15.20130344.

32. España MB, Zidane A, García JS, et al. Frecuencia de RX de tórax sugestivas de afectación por SARS-Cov-2 de marzo a mayo de 2020 en la población de un Área de Salud urbana. *Med Fam Semer* 2020; 2–5.

33. Shumilov E, Hosseini ASA, Petzold G, et al. Comparison of chest ultrasound and standard X-ray imaging in COVID-19 patients. *Ultrasound Int Open* 2020; 6: 36–40.

34. Bahreini F, Najafi R, Amini R, et al. Reducing False Negative PCR Test for COVID-19. *Int J Matern Child Heal AIDS* 2020; 9: 408–410.

35. España MB, Zidane A, García JS, et al. Frecuencia de RX de tórax sugestivas de afectación por SARS-Cov-2 de marzo a mayo de 2020 en la población de un Área de Salud urbana. *Med Fam Semer*. Epub ahead of print November 2020. DOI: 10.1016/j.semerg.2020.10.005.

36. Brogi E, Bignami E, Sidoti A, et al. Could the use of bedside lung ultrasound reduce the number of chest x-rays in the intensive care unit? *Cardiovasc Ultrasound*; 15. Epub ahead of print 2017. DOI: 10.1186/s12947-017-0113-8.

37. Mongodi S, Orlando A, Arisi E, et al. Lung Ultrasound in Patients with Acute Respiratory Failure Reduces Conventional Imaging and Health Care Provider Exposure to COVID-19. *Ultrasound Med Biol* 2020; 46: 2090–2093.

38. Andersen CA, Holden S, Vela J, et al. Point-of-care ultrasound in general practice: A systematic review. *Ann Fam Med* 2019; 17: 61–69.
