# Supplementary material for: Higher Accuracy of Lung Ultrasound over Chest X-ray for Early Diagnosis of COVID-19 Pneumonia
Source: Int J Environ Res Public Health. 2021 Mar 27;18(7):3481. doi: 10.3390/ijerph18073481 (PMC8037158; doi:10.3390/ijerph18073481)
Supplement: Supplementary file 1 [file ijerph-18-03481-s001.zip › supplementary files/Front Matter..pdf]

TITLE: Higher accuracy of lung ultrasound over chest X-ray for early COVID-19 diagnosis

Javier Martínez Redondo, Carles Comas Rodríguez, Jesús Pujol Salud, Montserrat Crespo Pons, Cristina García Serrano, Marta Ortega Bravo, Jose María Palacín Peruga

Javier Martínez Redondo, MD

General Practioner Balaguer Primary Care Center, Institut Català de la Salut (ICS), Lleida, Spain

Carles Comas Rodríguez, PhD

Mathematician, Department of Mathematics, Campus Cappont, University of Lleida (UdL) Lleida, Spain

Jesús Pujol Salud, PhD

General Practioner, Balaguer Primary Care Center, Institut Català de la Salut (ICS), Lleida, Spain

General Practioner, Universitat de Lleida (UdL). Biomedical Research Institute (IRB Lleida), Lleida, Spain

Montserrat Crespo Pons, BSc

Nurse, Balaguer Primary Care Center, Institut Català de la Salut (ICS), Lleida, Spain

Cristina García Serrano, BSc

Nurse, Balaguer Primary Care Center, Institut Català de la Salut (ICS), Lleida, Spain

Nurse, Research Group in Therapies in Primary Care (GRETAPS), Lleida, Spain

Marta Ortega Bravo, PhD

General Practioner, Research Group in Therapies in Primary Care (GRETAPS), Lleida, Spain

General Practioner, Research Support Unit Lleida, Fundació Institut Universitari per a la recerca a l'Atenció Primària de Salut Jordi Gol i Gurina (IDIAPJGol), Lleida, Spain

Jose María Palacín Peruga, MD

General Practioner, Onze de Setembre Primary Care Center, Institut Català de la Salut (ICS), Lleida, Spain.

***Corresponding authors:**

Jose María Palacín Peruga, MD
Onze de Setembre Primary Care Center, Institut Català de la Salut (ICS)
Passeig Onze de Setembre, 10, Lleida, 25005, Spain.
jpalacinp.lleida.ics@gencat.cat

Marta Ortega Bravo, PhD
Research Support Unit Lleida
Rambla Ferran, 44, Lleida, 25007, Spain.
mortega.lleida.ics@gencat.cat

Abstract

Background: The COVID-19 pandemic rapidly strained healthcare systems worldwide. The reference standard for diagnosis is a positive reverse transcription polymerase chain reaction (RT-PCR) test, but results are not immediate, and sensibility is variable.
Aim: To evaluate the diagnostic accuracy of lung ultrasound compared to chest X-ray for COVID-19.
Design and Setting: A retrospective analysis of symptomatic patients admitted into one primary care centre in Spain between March and September 2020.
Method: Patients' chest X-rays and lung ultrasounds were categorised as normal or pathologic. RT-PCR confirmed COVID-19 infection. Pathologic lung ultrasound images were further categorised as showing either local or diffuse interstitial disease. McNemar and Fisher tests were used to compare diagnostic accuracy.
Results: Most of the 212 patients presented fever at admission, either as a standalone symptom (37.74% of patients) or together with others (72.17% of patients). The positive predictive value of lung ultrasound was 90% for diffuse interstitial pattern and 46.92% for local pattern. Lung ultrasound had a significantly higher sensitivity (82.75%) ($P<0.001$), but lower specificity (71%) than chest X-ray (54.02% and 86%, respectively) ($P=0.008$) for identifying interstitial lung disease. Moreover, the sensitivity of lung ultrasound for severe interstitial disease was 100%, and was significantly higher than chest X-ray (58.33%) ($P=0.002$).
Conclusion: Lung ultrasound is more accurate than chest X-ray for identifying patients infected with COVID-19 and it is especially useful for those presenting diffuse interstitial disease.

Keywords: general practice, lung ultrasonography, thoracic radiography, COVID-19
